# The Effect of Right Temporal Lobe Gliomas on Left and Right Hemisphere Neural Processing During Speech Perception and Production Tasks

**DOI:** 10.3389/fnhum.2022.803163

**Published:** 2022-05-16

**Authors:** Adam Kenji Yamamoto, Ana Sanjuán, Rebecca Pope, Oiwi Parker Jones, Thomas M. H. Hope, Susan Prejawa, Marion Oberhuber, Laura Mancini, Justyna O. Ekert, Andrea Garjardo-Vidal, Megan Creasey, Tarek A. Yousry, David W. Green, Cathy J. Price

**Affiliations:** ^1^Neuroradiological Academic Unit, Department of Brain Repair and Rehabilitation, UCL Queen Square Institute of Neurology, University College London, London, United Kingdom; ^2^Lysholm Department of Neuroradiology, National Hospital for Neurology and Neurosurgery, London, United Kingdom; ^3^Neuropsychology and Functional Imaging Group, Departamento de Psicología Básica, Clínica y Psicobiología, Universitat Jaume I, Castellón de La Plana, Spain; ^4^Wellcome Centre for Human Neuroimaging, UCL Queen Square Institute of Neurology, University College London, London, United Kingdom; ^5^FMRIB Centre and Jesus College, University of Oxford, Oxford, United Kingdom; ^6^Faculty of Medicine, Collaborative Research Centre 1052 “Obesity Mechanisms”, University Leipzig, Leipzig, Germany; ^7^Department of Neurology, Max Planck Institute for Human Cognitive and Brain Sciences, Leipzig, Germany; ^8^Faculty of Health Sciences, Universidad del Desarrollo, Concepcion, Chile; ^9^Experimental Psychology, University College London, London, United Kingdom

**Keywords:** gliomas, fMRI, language, speech perception, speech production, right temporal lobe, neurosurgery

## Abstract

Using fMRI, we investigated how right temporal lobe gliomas affecting the posterior superior temporal sulcus alter neural processing observed during speech perception and production tasks. Behavioural language testing showed that three pre-operative neurosurgical patients with grade 2, grade 3 or grade 4 tumours had the same pattern of mild language impairment in the domains of object naming and written word comprehension. When matching heard words for semantic relatedness (a speech perception task), these patients showed under-activation in the tumour infiltrated right superior temporal lobe compared to 61 neurotypical participants and 16 patients with tumours that preserved the right postero-superior temporal lobe, with enhanced activation within the (tumour-free) contralateral left superior temporal lobe. In contrast, when correctly naming objects (a speech production task), the patients with right postero-superior temporal lobe tumours showed higher activation than both control groups in the same right postero-superior temporal lobe region that was under-activated during auditory semantic matching. The task dependent pattern of under-activation during the auditory speech task and over-activation during object naming was also observed in eight stroke patients with right hemisphere infarcts that affected the right postero-superior temporal lobe compared to eight stroke patients with right hemisphere infarcts that spared it. These task-specific and site-specific cross-pathology effects highlight the importance of the right temporal lobe for language processing and motivate further study of how right temporal lobe tumours affect language performance and neural reorganisation. These findings may have important implications for surgical management of these patients, as knowledge of the regions showing functional reorganisation may help to avoid their inadvertent damage during neurosurgery.

## Introduction

This study investigates how right temporal lobe gliomas, a type of primary brain tumour, alter the neural networks supporting speech perception and production tasks. Typically, gliomas present with epileptic type seizures rather than speech impairment (Laws et al., 1984; Chang et al., 2008; Ius et al., 2012), particularly in right handed patients who have tumours in the right cerebral hemisphere and so it is often assumed that the right cerebral hemisphere is less important for language function. Although early patient studies found that right hemispheric tumours do result in subtle language impairments (Gainott et al., 1981; Thomson et al., 1998), much of the previous research over the past 20 years has focused on tumours affecting the left hemisphere. Nevertheless, functional imaging of the healthy brain has increasingly shown that the right superior temporal lobe responds to human sounds (Belin et al., 2000), voice processing (Kriegstein and Giraud, 2004; Lattner et al., 2005; Andics et al., 2010), auditory feedback during speech production (Tourville et al., 2008; Behroozmand et al., 2015; Yamamoto et al., 2019b) and speech comprehension (Gajardo-Vidal et al., 2018). One region of the right superior temporal lobe in particular, the posterior superior temporal sulcus (RpSTS), is activated during both speech perception and production tasks and we have recently proposed that it is involved in integrating auditory expectations with auditory input (Yamamoto et al., 2019b).

Here we test the causal relevance of the right superior temporal lobe, in particular the RpSTS, for supporting speech perception and production, in patients with gliomas affecting this region. First we assess whether these patients have impaired language. Second, using fMRI, we investigate how the tumour may have altered the functioning of the neural networks during specific language tasks: auditory semantic matching (a speech perception task) and object naming (a speech production task). Brain injury occurring due to a tumour offers a unique insight into brain plasticity, as the potential for functional recovery is far greater with slow growing tumours than after an acute-onset insult to the brain such as occurs with a stroke (Desmurget et al., 2007). The rationale for our approach is as follows: if regions within the right superior temporal lobe typically support accurate and efficient speech processing then perturbed function resulting from the glioma will manifest as detectable performance deficits on detailed language assessment unless other brain areas, such as in the contralateral hemisphere, are able to support the function as has been shown in patients harboring left hemispheric lesions (Thiel et al., 2006, 2005; Krieg et al., 2013). In the current study three patients all with right temporal lobe gliomas of differing grades (grade 2, 3, 4) were assessed using a detailed language assessment to identify whether any had subtle language impairments which may not have been evident on clinical neurological examination performed at the bedside. A prior study (Noll et al., 2016), reported the results of three language tests administered to patients with right temporal lobe gliomas. Impaired performance was noted for 42% on verbal memory (Hopkins Verbal Learning Test), 10% on phonemic (letter) fluency and 7% on object naming; and these impairments were noted to be less severe than those reported in patients with left temporal lobe gliomas. Although there is less evidence that glioma grade influences performance on language tasks in patients (like ours) who have right hemisphere gliomas (Antonsson et al., 2018), prior studies have reported that cognitive and language impairments are more common (Bizzi et al., 2012), more severe (Zhang et al., 2018) or both (Noll et al., 2015a) for patients with left hemispheric high grade gliomas (HGGs) compared with low grade gliomas (LGGs). We therefore expected that language abilities would differ across our three patients given their different glioma grades.

We then performed fMRI to investigate the functioning of the speech perception and speech production networks in each of the three patients by comparing task related activation to that observed in 61 neurotypical control participants. This involved identifying: (a) any regions that the patients activated significantly more than the control participants when performing the same language task (Thiel et al., 2006) and (b) how such effects depended on the task. Enhanced activation in the patients compared to the control participants could either be within (intra-tumoural) or close to (peri-tumoural) the tumour or it could occur distant to the damage either within the ipsilateral or contra-lateral hemisphere (Duffau, 2005; Thiel et al., 2006; Desmurget et al., 2007). For example, the patients with right temporal lobe tumours might have abnormally high activation within or around the tumour-infiltrated right temporal lobe or they might have increased activation in homologue areas within the contralateral left superior temporal lobe (Thiel et al., 2005; Turkeltaub et al., 2011).

We focused on changes in task-specific brain activation that replicated across the three patients with right temporal lobe gliomas. Finally, we investigated whether these changes were: site-specific, pathology specific, both or neither by conducting the same investigations on three other patient groups: 16 patients with gliomas not involving the right postero-superior temporal lobe, eight stroke patients with right hemisphere infarcts affecting the right postero-superior temporal lobe and eight stroke patients with right hemisphere infarcts sparing it.

## Materials and Methods

### Participants

A total of 96 adults participated in an ethics committee approved fMRI study at the UCL Queen Square Institute of Neurology and the National Hospital for Neurology and Neurosurgery. 19 were patients, prospectively enrolled following referral for further management of their brain tumour. In 18 of these the diagnosis of glioma (WHO grade 1–4) was confirmed following surgery. 16 were patients who had previously suffered a right hemisphere infarct and were enrolled in the fMRI study after behavioural assessment and brain imaging for the PLORAS study (Seghier et al., 2016) confirmed that they were able to perform the fMRI tasks with high accuracy. The remaining 61 participants were neurologically intact (neurotypical control participants: NC). The patients were assigned to one of four groups:

#### Glioma Patients of Interest

**FIGURE 1:**
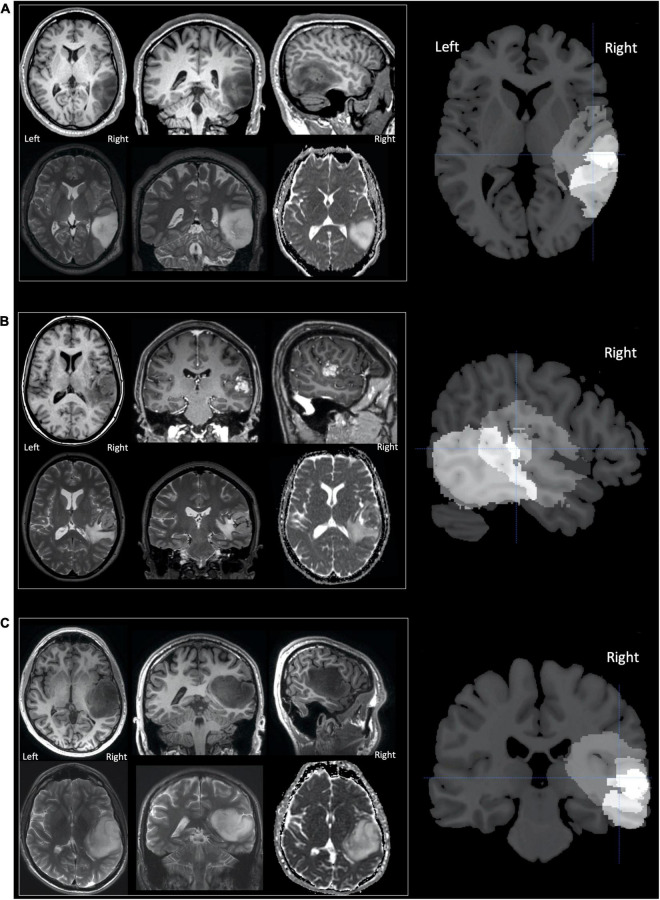
Tumours in patients **(A–C)**. Selected MR images for patients **(A–C)** are shown in neurological convention, with the right of the image corresponding to the right of the patient. The upper row, for each patient, displays (from left to right) axial, coronal and sagittal T1-weighted images. For patients A and C only unenhanced sequences are shown as their tumours were both non-enhancing. For patient B the coronal and sagittal images T1-weighted images are contrast enhanced and display a central enhancing component. The lower row for each patient (from left to right), displays axial and coronal T2-weighted images and the axial apparent diffusion coefficient (ADC) image from the diffusion weighted imaging (DWI). The right column of images shows sections from the lesion overlap map demonstrating the three tumours superimposed on one another—brighter voxels indicate more overlap between the three tumours. The overlap was greatest in the region of the superior temporal sulcus with the cross hair centred at [+51 −25 +5] which showed significantly altered activation during the speech perception and production tasks (see section “Results”).

Three adult neurosurgical patients (two male, one female) were the only patients enrolled in this time-limited fMRI study who had circumscribed tumours in the right postero-superior temporal lobe that infiltrated the right pSTS region that we have previously identified as involved in speech perception and production (Yamamoto et al., 2019b; see lesion overlap map in Figure 1). We refer to these patients as A, B and C, with A studied first and C studied last. The histological diagnoses of these patients are described below with demographic data provided in Table 1.

**TABLE 1:** Participant details.

Neurotypical control participants (NC)	Group 1 (MRI 1)	Group 2 (MRI 2)	
Number	51	10	
Gender (n female/n male)	31/20	7/3	
Median age in years (range)	37.1 (20–75.6)	23 (20.3–28.5)	
Handedness (right/left)	51/0	5/5	

**RpSTS glioma patients (GOI) (MRI 2)**	**A**	**B**	**C**

Age	25.5	41.2	19.8
Gender	M	F	M
Handedness	R	L	R
Clinical presentation	Seizure	Seizure	Seizure
Tumour type	OA (2)	GBM (4)	AA (3)
Tumour volume/cm^3^	60	11	97

**Glioma control patients (GC) (MRI 2)**			

Number	16		
Gender (n female/n male)	8/8		
Median age in years (range)	42.6 (21.6–58.5)		
Handedness (right/left)	13/3		

**Stroke patients (MRI 1)**	**RpSTS damage (SOI)**	**RpSTS preserved (SC)**	

Number	8	8	
Gender (n female/n male)	2/6	2/6	
Median age in years (range)	61.5 (45–73)	54.5 (26–72)	
Handedness (right/left)	7/1	8/0	
Median Years post stroke (range)	5.0 (1.9–14.2)	3.9 (0.8–15.4)	

*Demographic and clinical data for the different participant groups. MRI 1, imaged at location 1; MRI 2, imaged at location 2. Gender F, female; M, male. Handedness R, right; L, left. Tumour type (with WHO histological grading in brackets): OA, oligoastrocytoma; GBM, glioblastoma; AA, anaplastic astrocytoma (based upon the 2016 criteria; Louis et al., 2016). Tumour volume is in cm^3^ with measurement performed using manual segmentation in ITK-SNAP 3.6.0 (Yushkevich et al., 2006) by a neuroradiologist (author A.K.Y.).*

#### Glioma Control Patients

All other patients with supratentorial gliomas were assigned to this group (*n* = 16). One patient in this group did not proceed to surgery however they were included because imaging appearances of their tumour were consistent with a glioma. The histological diagnoses of these patients and additional demographic data are provided in Supplementary Appendix 1.

#### Stroke Patients of Interest

This group included eight patients with right hemisphere infarcts that (i) involved the right postero-superior temporal lobe and (ii) had activation in the F-map (*p* < 0.001) in the right postero-superior temporal region that we have previously identified as involved in speech perception and production (Yamamoto et al., 2019b). An additional four patients with right posterior temporal lobe damage participated in the fMRI paradigm but were not included in the current study because there was no residual parenchyma evident on MRI or no fMRI response (F-map from first level analysis thresholded at *p* < 0.001 uncorrected) in the region of interest within the right postero-superior temporal lobe. Demographic details for the eight included patients are provided in Table 1. An anatomical description of each infarct site is provided in Supplementary Appendix 2. Structural MR images, and the fMRI response are shown for each patient in Supplementary Appendix 3.

#### Stroke Control Patients

This group (*n* = 8) included the stroke patients with right hemisphere infarcts that preserved the right postero-superior temporal lobe according to (i) structural imaging and (ii) the F-map from the first level fMRI analysis (see Table 1 and Supplementary Appendix 2 for further details).

### Clinical Details of Patients of Interest With Right Postero-Superior Temporal Gliomas

Both patient A and patient C’s tumours were discovered on imaging shortly after the onset of generalised seizures. Patient B’s tumour was discovered incidentally on brain imaging performed 3 years previously for a different indication and had been subsequently monitored with imaging and shown to be gradually increasing in size. The onset of generalised seizures during this period of observation had resulted in referral to our institution. All patients were seizure free at the time of study participation (3 weeks for patient A, 9 months for patient B and 15 months for patient C) and were on anticonvulsant medication (levetiracetam for patient A, lamotrigine for patient B, sodium valproate and topiramate for patient C). None of the patients had previously received or were receiving treatment with corticosteroids at the time of the study.

Patients A and C were right handed. Patient B was left handed but we found no evidence that Patient B had right lateralised language responses. To the contrary, their language fMRI activation was entirely consistent with that observed in the two right handed patients. As is standard practice in our institution an experienced neuroradiologist provided a clinical fMRI report on two additional fMRI tasks which are acquired as part of standard clinical management. These were reported as being in keeping with left hemisphere dominance for all three patients.

None of the three patients reported any motor or visual symptoms. Patient B self-reported subtle changes in auditory function such as difficulty holding simultaneous conversations and understanding speech in the presence of other auditory stimuli. Patient C self-reported a decline in memory and concentration following diagnosis. None of the patients complained of difficulties with speech production.

Patient A’s tumour was centred on the middle temporal gyrus (MTG) and inferior temporal gyrus with the tumour extending to the superior temporal sulcus and medially into the deep and periventricular white matter bordering the lateral ventricle (Figure 1). Patient B’s tumour was the smallest, centred on the superior temporal lobe involving the superior temporal gyrus (STG) and planum temporale (PT) immediately adjacent to Heschl’s gyrus (HG). Unlike patients A and C, patient B’s tumour was surrounded by confluent white matter signal abnormality, which extended further into the anterior temporal lobe, medially to the periventricular white matter and superiorly into the inferior parietal lobule. Patient C’s tumour was the largest and was also centred on the right superior temporal lobe involving HG, PT and STG. Medially, the tumour extended into the posterior insula.

Based upon the World Health Organization 2016 classification (Louis et al., 2016) which was in use at the time of this study, the histological diagnosis of glioma was confirmed in patient A (WHO grade 2 oligoastrocytoma IDH mutant, 19q deletion), patient B (WHO grade 4 glioblastoma IDH wildtype) and patient C (WHO grade 3 anaplastic astrocytoma IDH mutant). The volume of each patient’s tumour ranged from 11 to 97cm^3^ (see Figure 1). The structural imaging performed in patients A and C demonstrated non-enhancing tumours. Patient’s B tumour demonstrated enhancement consistent with higher grade features at the time of study though the tumour had been present for at least 3 years prior to referral. Patients A and B underwent surgery shortly afterward in our institution, this was performed in the intra-operative MRI surgical suite. Given the pre-operative clinical fMRI findings of left hemispheric dominance, both surgeries were performed under general anesthesia without intra-operative language mapping. Patient C underwent surgery in a different institution, this was performed after one further year of observation.

### Language Testing

Each patient (glioma and stroke) underwent behavioural testing performed by a speech and language therapist using the Comprehensive Aphasia Test (CAT) (Swinburn et al., 2004) which consists of twenty one language related tests and six cognitive non-language tasks taking approximately 1–2 h to complete, depending on ability. Each task generates raw scores which are expressed as T-scores relative to a reference group of 113 aphasic patients and 27 controls. This classifies each patient’s performance as being either normal or impaired. Lower scores indicate lower performance.

### fMRI

#### Experimental Design

All participants (all patients and NC) performed the same language fMRI paradigm with five different conditions that have previously been described (Sanjuán et al., 2015) with further examples of the fMRI tasks illustrated in Ekert et al. (2021). We report data from three tasks. The first task required participants to listen to pairs of spoken object names that were either semantically related (e.g., pizza and tomato) or unrelated (e.g., fridge and camera), recognise the spoken objects (speech perception), hold these names in auditory short-term memory and press a button to indicate if the two objects referred to were semantically related or not. The index finger on the dominant hand was used if the objects were semantically related and the middle finger of the same hand was used if the two objects were semantically unrelated. The second task involved naming aloud two objects in each of a series of pictures and also linking these with “and” (e.g., “cup and piano”). The third task was a control condition that involved seeing pictures of two objects (as in Task 2) and semantically matching them (as in Task 1).

Semantically related items were either from the same category (e.g., elephant and giraffe, grapes and pear, harp and piano) or the same location (e.g., dolphin and sea; mermaid and rocks; crab and shell). Half the stimuli in Tasks 1 and 3 presented semantically related objects. None of the object pairs used for object naming (Task 2) were semantically related. The two remaining conditions presented pictures of two objects interacting with one another (e.g., camera falling off the chair); and required either the production of a full sentence (“the camera is falling off the chair”) or the verb only (“falling”). We did not include these conditions in the current study because they were not matched, for content, to the visual and auditory semantic matching conditions. However, we note here that there was (a) no significant difference (*p* > 0.001) in activation for sentence production and object naming in any of the regions reported in this study and (b) significantly less activation in these regions for verb naming compared to object naming. This was expected given that only 4 verbs were used (eating, drinking, jumping, falling) and each response involved only one of these words (as opposed to two object names).

Different stimuli were presented in different conditions, but there was no rotation of stimuli across conditions in different participants and there was no counterbalancing of conditions across participants. This is because the aim of the study was to test how brain responses differ across participants and to interpret inter-subject differences, we therefore needed to hold all other experimental details (stimuli and task order) constant across participants. All participants (patients and controls) were each presented with five conditions in the following order: (1) visual semantic matching on pictures of two objects (our baseline task); (2) naming the objects in pictures (our speech production task); (3) naming the verb in a picture of an event; (4) producing a sentence to describe the event in a picture; and (5) auditory semantic matching on two heard object names (our speech perception task).

#### Stimulus Selection/Preparation

Across the experiment, each participant was presented with 120 animal or object concepts, either in picture format or as spoken speech. The names associated with these objects had one to four syllables in English (mean 1.59; standard deviation (SD) 0.73). To ensure that all participants recognised the objects and produced highly accurate behavioural responses, a prior pilot study was conducted on 26 young healthy adults and inter-subject variability was evaluated in (i) the name associated with each object and animal, and (ii) whether two objects were considered to be semantically related. On the basis of the pilot results, all the objects/animals that were most consistently named during object naming were assigned to the object naming condition. From the remaining stimuli, those with the strongest semantic associations were assigned as related pairs in the semantic matching conditions (visual or auditory). The remaining stimuli were assigned as unrelated pairs.

#### Stimulus Presentation

Each task was presented in a separate scan run with four blocks of five object pairs, presented at a rate of one every 5 seconds (s) (i.e., 10 objects in 25s blocks). Every stimulus block was preceded by a written instruction (e.g., “Name objects”), lasting 3.08s (equivalent to one interscan interval), which indicated the start of a new block and reminded subjects of the task. Within each semantic matching block (auditory and visual), there were two or three semantically related pairs and three or two semantically unrelated pairs.

All visual stimuli (pictures of objects) were presented via an LCD projector, and an adjustable head-coil mirror, onto a screen that was clearly visible to the subject. Together they subtended a visual angle of 7.4 degrees with 1,024 × 768 resolution after scaling to 350 × 350 pixels. The duration of each visual stimulus was 2.5 s, with 3.5 s fixation between stimuli (i.e., 5 s interstimulus interval).

All auditory stimuli were presented for 1.76–2.5 s via MRI compatible headphones (MR Confon, Magdeburg, Germany), which filtered ambient in-scanner noise. The mean duration of the auditory stimuli (two object names linked with “and”) was 1.92 s from the onset of the first word to the offset of the second word (standard deviation of 0.13 s). Volume levels were adjusted for each subject before scanning. During stimulus presentation, participants fixated on a cross centred on the screen to control.

Each stimulus block was followed by 16 s resting periods, during which participants fixated on a cross centred on the screen. These resting with fixation periods allowed activation to return to baseline between blocks, therefore ensuring maximum sensitivity to all effects of interest.

#### Behavioural Response Acquisition

Prior to scanning, all participants were instructed as to the task requirements and familiarised with the procedure using pictures and auditory stimuli which were separate to those being used in the scanner. During scanning, participants were reminded to keep their eyes open for all tasks, including the auditory task. They were also frequently reminded to remain as still as possible to prevent movement-induced acquisition noise.

Spoken responses were recorded for each subject via a noise-canceling MRI microphone (FOMRI III™ Optoacoustics, Or-Yehuda, Israel), and transcribed manually for off-line analysis. For auditory and visual semantic decision tasks, participants used two fingers of the same hand (left hand for 15 subjects) to press one of two buttons on an fMRI compatible button box.

#### Behavioural Data Processing

Responses to each stimulus were transcribed at the time of imaging and scored for accuracy off-line by listening to the audio files (for object naming) and by reviewing the recorded data from the button press tasks. Each response was categorised as correct (when the response matched the target) or incorrect. If >10% of participants across the study produced a spoken response that did not match the target but did match the meaning (e.g., target = mug, response = cup), responses for these participants were also marked as “correct.” Other (i.e., atypical responses) were classified as “incorrect.”

Reaction times (RTs) for spoken responses were obtained from the audio files. To compute them, we used an adaptive moving window filter that was tailored to each subject. The optimal window length (i.e., the width which maximally smoothed the audio stream) was based on a portion of the audio file collected during rest. After smoothing the whole time series, we defined the onset of speech as a rise in the absolute amplitude of the smoothed audio stream beyond 1.5 standard deviations from the mean.

Accuracy and response times (from stimulus onset to response) were successfully collected for all trials for all participants except for one missing response time for visual semantic matching of one participant in the NC group; five missing responses at the end of visual semantic matching (the control task) for Patient C; and 11 missing response times from Patient C during object naming because of technical failures in the voice recording system. When semantic matching responses were missing (six in total), we could not be sure they were correct, therefore these trials were excluded from the behavioural and also fMRI statistical analyses that focused on correct responses using an event-related analysis. Accuracy during object naming was recorded by listening to participant responses during scanning and this indicated that all the object naming trials with missing response times were accurate. These trials were therefore only excluded from the analysis of in-scanner response times and not from the accuracy or fMRI analyses. Although loss of data points can influence the sensitivity of statistical contrasts, it has no bearing on the interpretation of significant effects that replicate across all three of our patients.

#### MRI Acquisition

Functional and structural research sequences were performed using the same acquisition technique on 3T Siemens scanners (Erlangen, DE) with 12-channel head coils. Data were acquired at two different locations: The Wellcome Centre for Human Neuroimaging (location 1) and the National Hospital for Neurology and Neurosurgery (location 2). Location 1 was used for 51 of the NC group and all the stroke patients (*n* = 16); location 2 was used for the remaining 10 of the NC group and all the patients with gliomas (*n* = 19). Total scanning time was approximately 1 h and 30 min per subject, including set up and the acquisition of a structural scan.

Functional images for all participants were acquired using a gradient echo (GE) echo planar imaging (EPI) sequence with in plane resolution = 3 × 3 mm^2^, repetition time TR = 3,080 ms, echo time TE/flip angle = 30 ms/90°, field of view (FOV) = 192 × 192 mm^2^ and matrix size = 64 × 64. The TR (3,080 ms) was chosen to maximise whole-brain coverage and allowed us to asynchronise the slice acquisition with stimulus onset (5,000 ms) to allow for distributed sampling of stimulus onsets across slices in each condition (Veltman et al., 2002). For example, when the first stimulus in a block was presented at the start of the TR (slice 1 of 44), subsequent stimuli were presented at slices: 11, 22, 27, and 38.

For locations 1 and 2 respectively, slice thickness = 2 mm/2.5 mm, interslice gap = 1 mm/0.5 mm, number of slices = 44/49 (for whole brain coverage), volumes per time series within run = 66/64, with 5/3 dummy scans (to allow for T1 equilibration effects).

At location 1, T1-weighted anatomical images were acquired after the EPI sequences, using a 3-D modified driven equilibrium Fourier transform sequence (TR/TE/TI/flip angle = 7.92 ms/2.48 ms/910 ms/16°, voxel size = 1 × 1 × 1 mm^3^,176 slices). At location 2, T1-weighted anatomical images were acquired with a magnetisation prepared rapid acquisition gradient echo (MPRAGE) sequence (Mugler and Brookeman, 1990) in the sagittal plane (TR/TE/TI/flip angle = 2,200 ms/2.88 ms/900 ms/10°, voxel size = 1.1 × 1.1 × 1.1 mm^3^, 176 slices). Clinical MRI sequences were performed as part of routine pre-neurosurgical management.

#### fMRI Data Pre-processing

We performed the fMRI data analysis in SPM12 (Wellcome Centre for Human Neuroimaging, London, United Kingdom), running on MATLAB 2012a (MathWorks, Natick, MA, United States). Functional volumes were (a) spatially realigned to the first EPI volume and (b) un-warped to compensate for non-linear distortions caused by the interaction between head movement and magnetic field inhomogeneity. The anatomical T1-weighted image was (c) co-registered to the mean EPI image which had been generated during the realignment step and then spatially normalised to the Montreal Neurological Institute (MNI) space using the new unified normalisation-segmentation tool of SPM12. To spatially normalise all EPI scans to MNI space, (d) we applied the deformation field parameters that were obtained during the normalisation of the anatomical T1 image. The original resolution of the different images was maintained during normalisation (voxel size 1 × 1 × 1 mm^3^ for the structural T1-weighted and 3 × 3 × 3 mm^3^ for the EPI images). Due to the presence of the right temporal tumours, the co-registration and normalisation images were carefully reviewed by a neuroradiologist (A.K.Y) to ensure that there were no mis-registrations apparent to the eye. After the normalisation procedure, (e) functional images were spatially smoothed with a 6 mm full-width-half-maximum isotropic Gaussian Kernel to compensate for residual anatomical variability and to permit application of Gaussian random-field theory for statistical inference (Friston et al., 1995).

#### First Level Statistical Analyses

In the first-level statistical analyses, each pre-processed functional volume was entered into a subject specific fixed-effect analysis using the general linear model. An event-related analysis was performed, which allows for the modeling of brain responses for correct trials with the exclusion of incorrect trials (Mechelli et al., 2003). All stimulus onset times were modeled as single events, with only the correct response trials as regressors of interest and three extra regressors that included the instructions, incorrect or “other” (self-corrected, delayed or no-response) trials. Stimulus functions were then convolved with a canonical hemodynamic response function. To exclude low-frequency confounds, the data were high-pass filtered using a set of discrete cosine basis functions with a cut-off period of 128 s. The contrasts of interest compared each of the different conditions (correct trials only) to fixation.

#### Second Level Statistical Analyses

##### Second Level Analysis 1

To identify regions of interest, we compared the fMRI response for our three tasks of interest in the three patients with gliomas of interest (GOI) to the 61 NC. The patients were modeled as three independent groups (one patient per group) and the NC were modeled as two independent groups (51 scanned at location 1 and 10 scanned at location 2). We then identified group differences in activation for: (1) auditory semantic matching vs. rest, (2) visual object naming vs. rest, (3) auditory semantic matching vs. visual semantic matching, and (4) visual object naming vs. visual semantic matching. Contrasts (3) and (4) allowed us to focus on activation that was more likely to be related to speech perception and production respectively (after controlling for semantic and perceptual or motor responses).

We only report group differences that are (i) highly significant (*p* < 0.05 with family wise error correction across the whole brain) when comparing activation across the three patients to that across the two NC groups; (ii) also significant for each patient compared to the NC groups (using an uncorrected threshold of *p* < 0.05) and (iii) not sensitive to scanner differences (i.e., at location 1 or 2). The combination of statistical criteria that all needed to be satisfied to infer abnormal activation are detailed in Table 2.

**TABLE 2:** Statistical criteria and thresholds used for identifying abnormal activation.

Task:	Auditory semantic matching		Object naming	
Baseline:	>Rest	>Vis Sem Mat	>Rest	>Vis Sem Mat
Abnormally low activation				
(1) NC only	*p* < 0.05c	*p* < 0.05c	*p* < 0.05c	*p* < 0.05c
(2) NC > All 3 Patients	*p* < 0.05c	*p* < 0.05c	*p* < 0.05c	*p* < 0.05c
(3) NC > Patient A	*p* < 0.05u	*p* < 0.05u	*p* < 0.05u	*p* < 0.05u
(4) NC > Patient B	*p* < 0.05u	*p* < 0.05u	*p* < 0.05u	*p* < 0.05u
(5) NC > Patient C	*p* < 0.05u	*p* < 0.05u	*p* < 0.05u	*p* < 0.05u
**Abnormally high activation**				
(1) All 3 patients	*p* < 0.05c	*p* < 0.05c	*p* < 0.05c	*p* < 0.05c
(2) All 3 Patients > NC	*p* < 0.05c	*p* < 0.05c	*p* < 0.05c	*p* < 0.05c
(3) Patient A > NC	*p* < 0.05u	*p* < 0.05u	*p* < 0.05u	*p* < 0.05u
(4) Patient B > NC	*p* < 0.05u	*p* < 0.05u	*p* < 0.05u	*p* < 0.05u
(5) Patient C > NC	*p* < 0.05u	*p* < 0.05u	*p* < 0.05u	*p* < 0.05u

*The table lists the statistical criteria used to identify regions of abnormal activation in the glioma patients of interest (GOI) compared to the neurotypical control participants (NC) for auditory semantic matching > rest/visual semantic matching; and object naming > rest/visual semantic matching. Group effects (contrasts 1-2) were assessed at a statistical threshold of p < 0.05 with family wise error correction across the whole brain (p < 0.05c) and individual patient effect (contrasts 3–5) were assessed, within these regions, at an uncorrected threshold of p < 0.05 (p < 0.05u). Results are reported for contrast (2) to (5) in Tables 5A,B.*

##### Second Level Analysis 2

To identify site specific and pathology specific effects, the results from second level analysis 1 were further investigated in a *post hoc* analysis that included the first level contrasts for the auditory semantic matching and object naming tasks for all 96 participants. These two tasks were modeled separately for 5 groups: NC (*n* = 61), GOI (*n* = 3), GC (*n* = 16), SOI (*n* = 8) and SC (*n* = 8). Focusing on regions of interest from Second level analysis 1, we plot the data from each task for each participant and report the significance of the statistics for the interactions between task (object naming vs. auditory semantic matching) and group (GOI vs. GC and SOI vs. SC).

## Results

### The Effect of Right Posterior Superior Temporal Sulcus Damage on Standardised Language Scores

All three patients with RpSTS gliomas had non-aphasic scores on test of speech comprehension and object recognition including: word-to-picture matching, sentence-to-picture matching, sentence repetition, semantic matching (with pictures) and paragraph comprehension. However, they had reduced scores (in the mildly impaired range) when naming objects in pictures and when matching written words to pictures (a written word comprehension test). There was no evidence to suggest these impairments could be explained by difficulties in recognising pictures or semantic matching because all three patients had normal scores when matching pictures on the basis of their semantic relationships and when matching heard speech (spoken words and sentences) to complex pictures. Patient C also had impaired scores on repeating phonologically complex words, reading words aloud, and recognition memory (see Table 3). Importantly the overall scores between the three patients for written comprehension and object naming were very similar, with patient B with the grade 4 glioma no more impaired than patient A with the grade 2 glioma.

**TABLE 3:** CAT test results for patients with gliomas of interest.

CAT Test		Cut-off	A	B	C
Written comprehension	Words Ts	54	**50^†^**	**53^†^**	**51^†^**
	Raw score	27/30	**25**	**27**	**26**
Speech production	Reading words Ts	61	69	69	**57^†^**
	Raw score	45/48	48	48	**42**
	Object naming Ts	61	**61^†^**	**60^†^**	**61^†^**
	Raw score	43/48	**42**	**41**	**43**
	Repeating complex words Ts	61	62	62	**55^†^**
	Raw score	5/6	6	6	**5**
Cognitive	Recognition memory Ts	47	48	59	**32^†^**
	Raw score	8/10	9	10	**4**

*Results from the Comprehensive Aphasia Test (CAT) are shown for the subtests that were impaired for patients A, B and C. Raw scores are converted to T-scores following comparison to a reference population of 113 patients. If the score is equal or below the cut-off value, this is classified as impaired performance, marked by † in **bold**. The raw score indicates how many errors the patients made, or in the cut-off column, the total number of points available (denominator), and the highest number associated with impaired performance (numerator). The patients performed in the normal range for the remainder of the subtests from the CAT.*

For stroke patients with damage to RpSTS, we found that, like the patients with RpSTS gliomas none had impaired speech comprehension, object recognition or semantic matching. Mild impairments were recorded for: comprehension of written words (*n* = 2); repetition of words (*n* = 2) and repetition of non-words (*n* = 1). However, unlike the patients with RpSTS gliomas, none of the stroke patients with RpSTS damage had impaired object naming scores. Plausibly, this is because they were all tested years after their strokes (1.9–14.2 years) (see Table 1 and Supplementary Appendix 2).

### In Scanner Behavioural Results

All but three participants scored at least 75% correct on each task. The exceptions were one stroke control patient who scored 60% on the auditory semantic matching, one neurotypical control participant who scored 70% for object naming and another neurotypical control participant who scored 55% for visual semantic matching. Accuracy for patients of interest (with RpSTS gliomas or stroke patients) was within the normal range for each of the three tasks. Table 4 summarises the in scanner behavioural performance for the NC group and patients with RpSTS gliomas.

**TABLE 4:** In scanner behavioural performance for patients with RpSTS gliomas.

Task		NC group 1	NC group 2	A	B	C
Auditory matching	Accuracy	96 (4)	96 (6)	95	100	100
		[85–100]	[80–100]			
	RT	2,600 (203)	2,539 (194)	2,404	2,366	2,517
		[2,250–3,291]	[2,248–2,945]			
Object naming	Accuracy	94 (5)	91 (10)	90	80	100
		[80–100]	[70–100]			
	RT	1,310 (157)	996 (380)	1,126	1,107	n/a
		[1,006–1,917]	[426–1,803]			
Visual matching	Accuracy	96 (7)	99 (2)	95	100	93
		[55–100]	[95–100]			
	RT	1,287 (263)	1,131 (189)	1,180	1,262	1,037
		[860–2,212]	[881–1,386]			

*NC groups 1 and 2 refer to the two groups of neurotypical control participants (according to the location used for data acquisition). A, B, and C refer to the three patients with RpSTS gliomas. Accuracy is the mean percentage of correct responses for each task with (standard deviation) and [range]. RT is the response time in milliseconds with (standard deviation) and [range]. n/a = not applicable because the response times could not be collected for technical reasons as detailed in the Materials and Methods section.*

### fMRI Results

#### Auditory Semantic Matching (Second Level Analysis 1)

**FIGURE 2:**
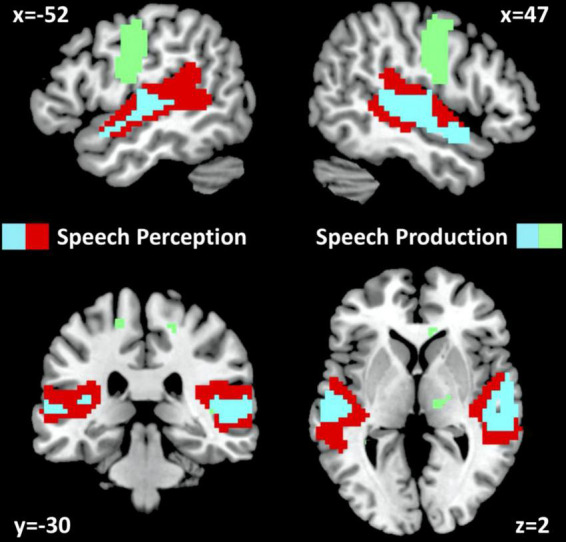
fMRI activation for neurotypical control participants. fMRI activation clusters are shown for the 61 neurotypical control participants (NC) for the speech perception task (auditory semantic matching > visual semantic matching) and the speech production task (object naming > visual semantic matching). Cyan represents activated voxels for both auditory semantic matching and object naming. Red represents activated voxels for auditory semantic matching only and green represents activated voxels for object naming only. The statistical threshold was *p* < 0.05 with family wise error correction across the whole brain.

In NC, auditory semantic matching increased activation (compared to rest and visual semantic matching) in bilateral superior temporal gyri (red and cyan regions in Figure 2) with no significant difference between those imaged in Location 1 or 2.

**FIGURE 3:**
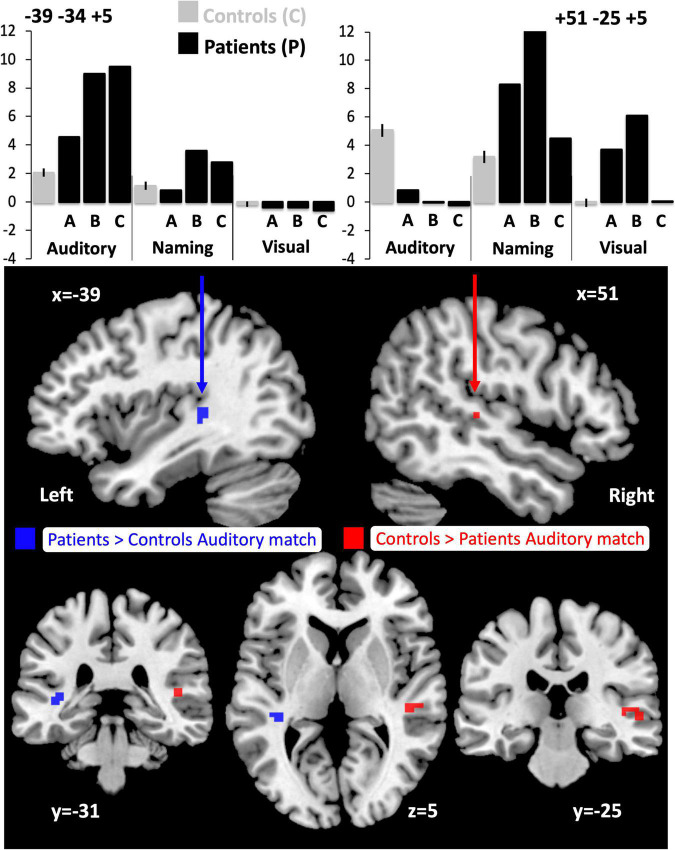
Altered responses during auditory semantic matching, object naming and visual semantic matching in the three RpSTS glioma patients compared with neurotypical control participants. The plots (top) illustrate the fMRI responses in the left [−39 −34 +5] and right [+51 −25 +5] superior temporal lobes for the three fMRI tasks: auditory semantic matching (auditory), object naming (naming) and visual semantic matching (visual). Y-axis represent the fMRI responses (first eigenvariate extracted from SPM). The images below show the sites of abnormal fMRI activation (*p* < 0.05 after family wise error correction across the whole brain) in the patients compared to the neurotypical controls participants (NC), overlaid on a normalised T1-weighted image in neurological convention (right of subject on right of image). Blue indicates the left superior temporal region where patients showed an enhanced response during auditory semantic matching (11 voxels). Red (in the right pSTS) shows where the patients under-activated during auditory semantic matching compared to NC (19 voxels).

Compared to the NC group, the three patients with right pSTS gliomas showed significantly less activation in the right superior temporal lobe (Table 5A). This was observed, for all three patients, in the region infiltrated by the tumours, sited between the right posterior superior temporal sulcus (pSTS) and right planum temporale (PT) (red cluster in Figure 3) with peaks at MNI [+51 −25 +5] and [+45 −28 +5]. In contrast, activation in the left homologue, bordering the left pSTS and left PT [−39 −34 +5], was higher (enhanced) in each of the patients compared to the controls (Table 5A, blue clusters Figure 3). These group and individual patient effects were seen for auditory semantic matching compared to both rest and visual semantic matching (Table 5A).

**TABLE 5:** Comparison of patients with RpSTS gliomas to neurotypical control participants.

(A) Auditory semantic matching				
**Contrast**	**Patients**	**NC > Patients**		**Patients > NC**

		**+51 −25 +5**	**+45 −28 +5**	**−39 −34 +5**
Auditory semantic	A, B, and C	**5.1**	**5.4**	**6.8**
matching > Rest	Patient A	2.7	3.2	1.9
	Patient B	3.2	3.5	5.1
	Patient C	3.3	3.0	5.4

Auditory > Visual	A, B, and C	**5.9**	**4.5**	**5.1**
semantic matching	Patient A	3.5	3.3	1.7
	Patient B	4.8	3.0	3.7
	Patient C	2.4	1.9	4.1

**(B) Object naming**				

**Contrast**	**Patients**	**Patients > NC**		

		**+51 −25 +5**	**+42 −34 −4**	**+39 −40 +11**

Object naming	A, B, and C	**5.2**	**5.5**	**7.9**
> Rest	Patient A	3.3	5.3	8.5
	Patient B	5.3	2.0	1.8
	Patient C	ns	2.8	2.7

Object naming	A, B, and C	ns	**4.9**	**7.4**
> Visual semantic matching	Patient A	1.8	3.9	7.9
	Patient B	3.8	1.9	2.7
	Patient C	ns	2.3	2.3

**(C) Task by group interactions**				

**Contrast**	**Patients**	**Patients > NC**		

		**+51 −25 +5**	**+45 −28 +5**	**−39 −34 +5**

Auditory semantic	A, B, and C	ns	ns	3.6
matching > Naming	Patient A	ns	ns	1.7
	Patient B	ns	ns	2.2
	Patient C	ns	ns	2.9

Naming > Auditory	A, B, and C	**7.1**	**5.3**	ns
semantic matching	Patient A	4.2	4.1	ns
	Patient B	5.9	3.3	ns
	Patient C	3.1	2.3	ns

*Group differences in fMRI activation, for patients with RpSTS gliomas (A, B, C) compared to 61 neurotypical control participants (NC) for: (A) auditory semantic matching, (B) object naming and (C) the interaction of auditory semantic matching and object naming. Co-ordinates (MNI) are from the comparison of all three patients (A, B, and C) to NC. Z-scores for individual patients are all within a 3 mm sphere of these co-ordinates. Statistical thresholds: Z scores ≥ 4.8 = p < 0.05 after family wise error correction across the whole brain (shown in bold), ≥ 3.1 = p < 0.001 uncorrected and ≥ 1.7 = p < 0.05 uncorrected. ns = not significant at p < 0.05 uncorrected. See Figure 3 and text for anatomical localisation.*

**FIGURE 4:**
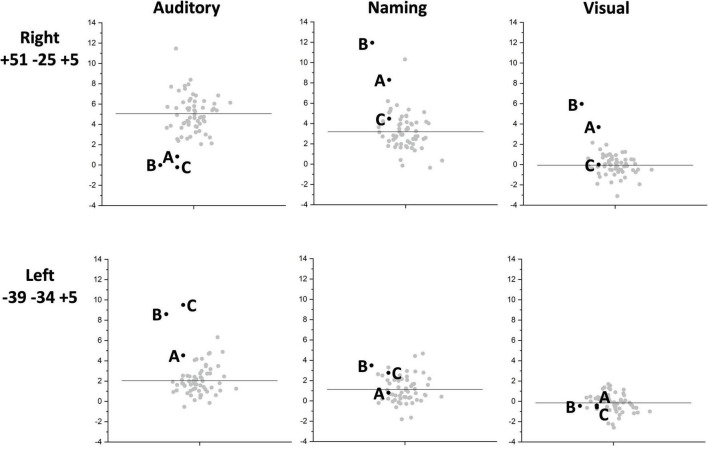
Inter-subject task variability plots for the right and left temporal regions of interest. The plots show the individual responses for the three patients (black circles) and 61 neurotypical control participants (grey circles) for the right superior temporal [+51 −25 +5] (upper row) and left superior temporal [−39 −34 +5] (lower row) regions showing group differences. These are shown for each of the three fMRI tasks: auditory semantic matching (Auditory) (left column plots), object naming (Naming) (centre column plots) and visual semantic matching (Visual) (right column plots). The black horizontal line indicates the average response across the 61 NC. Y-axis represent the fMRI responses (first eigenvariate extracted from SPM).

Individual responses, from all three patients with right pSTS gliomas and 61 controls, are plotted in Figure 4 to illustrate that each patient showed reduced and increased activation relative to the average control response within the right and left superior temporal lobes respectively. This effect is most notable at [+51 −25 +5] where all three patients’ responses were below the normal range. Outside the auditory cortices, there were no other significant abnormalities in activation during auditory semantic matching that were observed in each of the three patients.

#### Object Naming (Second Level Analysis 1)

In NC, visual object naming (compared to either rest or visual semantic matching) increased activation in bilateral superior temporal gyri, pre-central and post-central gyri and supero-inferior medial cerebellar and subcortical regions (green and cyan regions in Figure 2). The only activation difference for NC imaged at Location 1 or 2 was in bilateral cerebellum which is not relevant to the current results.

Across the three patients with right pSTS gliomas, there were no brain regions, including RpSTS that were consistently less activated than the neurotypical control participants. Even more surprisingly, patients showed higher than normal activation in RpSTS during naming where we found lower than normal activation during auditory semantic matching (see Table 5 and Figures 3, 4). Table 5C shows the significance of this task by group interaction, along with the opposite pattern of effects in the left superior temporal lobe where patient activation was significantly higher than normal during auditory semantic matching, but not during object naming (see plots in Figures 3, 4).

#### Other Patient Groups (Second Level Analysis 2)

**FIGURE 5:**
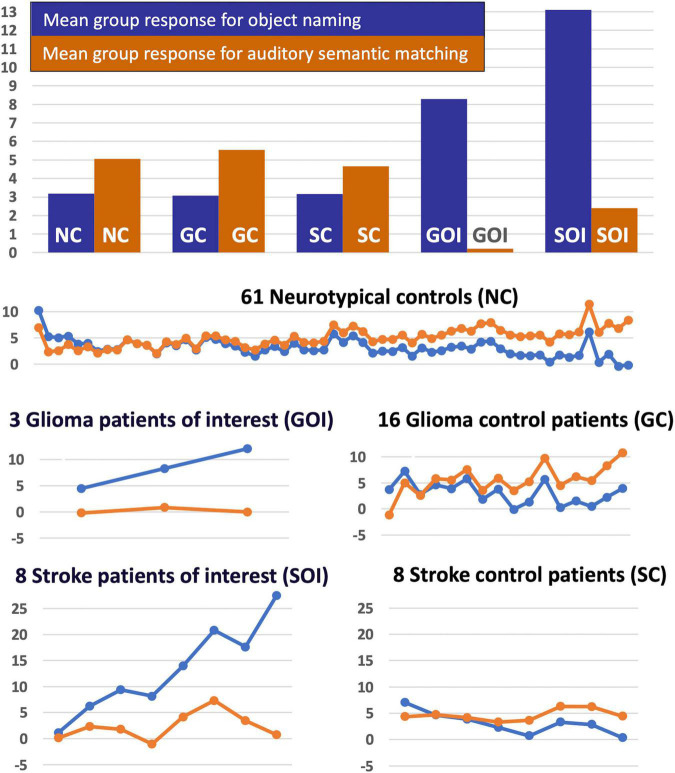
Task-specific fMRI response in RpSTS [+51 −25 +5]. The task-specific responses (blue = object naming, light brown = auditory semantic matching) in Right pSTS [+51 −25 +5] are shown for the five groups of participants (NC, GC, SC, GOI, and SOI). The uppermost chart shows the mean group response for the two tasks for each of the five groups. The lower plots show the individual responses for the two tasks for each participant in each of the five groups (in total *n* = 96). Y-axis represent the fMRI responses (first eigenvariate extracted from SPM).

Our final analysis demonstrated that the task dependent group differences in RpSTS (under-activation during auditory semantic matching and over-activation during object naming) were also observed when (i) our three patients with RpSTS gliomas were compared to 16 patients with gliomas that spared RpSTS and (ii) in the comparison of right hemisphere stroke patients with damage vs. preservation of RpSTS (Table 6). Figure 5 compares the group and individual responses at the peak RpSTS region [+51, −25, +5]. Almost all the control participants (61 neurotypical and 22 patients) show more or equivalent activation for auditory semantic matching (light brown) than object naming (blue). This pattern reverses for the three patients with RpSTS gliomas and the eight patients with (incomplete) RpSTS stroke damage.

**TABLE 6:** Enhanced right temporal lobe activation for object naming > auditory semantic matching.

	+51 −25 +5	+45 −28 +5	+39 −40 +11	+42 −34 −4
GOI > GC	4.6	3.5	4.3	2.2
SOI > SC	5.6	5.9	4.1	1.7

*Statistical significance (Z scores) of group differences in activation for object naming more than (>) auditory semantic matching at each of the right temporal lobe co-ordinates identified in the comparison of RpSTS gliomas to neurotypical control participants (see Table 5 for details). The results are shown for the comparisons of the Glioma patients of interest (GOI) > Glioma control patients (GC) (upper row) and the Stroke patients of interest (SOI) > Stroke control patients (SC).*

In the left temporal lobe, enhanced activation during auditory semantic matching was observed for our three glioma patients of interest (with RpSTS damage) compared to the 16 patients with gliomas that spared RpSTS (*Z* = 6.5; *p* < 0.05 corrected at −39, −34, +5), but activation in this region was not higher than normal in any of the 16 stroke patients during either task.

## Discussion

The aim of this study was to investigate if gliomas within the right postero-superior temporal lobe, involving speech sensitive language regions, result in language impairment and how they affect the functioning of the neural networks involved in the perception and production of speech. We searched for atypical neural activation that was common to patients with three differing grades of right temporal lobe gliomas (grade 2, 3, 4) compared to (i) neurotypical control participants and (ii) patients with gliomas in other supratentorial regions. We also tested whether the same effects were observed when comparing brain activation from patients with right hemisphere stroke damage that affected, or did not affect, the right postero-superior temporal lobe. This revealed cross-pathology effects that were task specific and site specific. Below, we describe the effect of RpSTS gliomas on speech and language abilities and the neural processing associated with speech perception and production tasks in the context of prior literature, before discussing the role of tumour plasticity, study limitations and clinical implications.

### The Effect of Right Posterior Superior Temporal Sulcus Damage on Speech and Language Behaviour

Despite having tumours that infiltrated the right temporal lobe regions implicated in speech perception and production, none of the three patients with RpSTS gliomas showed language impairments on clinical examination or speech perception impairments on standardised speech and language assessments. However, all three of these patients had mild impairments on tests of object naming and written comprehension. This finding complements and extends prior literature. For example, Noll et al. (2016) found that 2/29 (7%) of their patients with right temporal lobe gliomas had object naming impairments, 3/29 (10%) had letter fluency impairments and 12/30 (40%) had impaired verbal learning (total recall). Antonsson et al. (2018) also reported the pre-operative results of language assessment in a patient who had a tumour that infiltrated the temporal lobe (in addition to frontal, insula and thalamic regions) with this patient having below cut off scores on word definitions and comprehension of logico-grammatical sentences and sentence analysis but not object naming.

Variability in the effect of right temporal lobe damage on language function was also observed in our sample of eight patients with stroke damage to RpSTS, half of which had mild impairments with written comprehension and/or repetition of words/non-words. Further studies are required to understand whether the variability observed in our own data and prior studies is the consequence of: the degree and location of damage within the right temporal lobe and the extent of damage to other brain regions, the histological grading and speed of growth of the tumours and the degree of functional re-organisation of the language networks which is likely to depend on pathology duration.

### Neural Processing Associated With Speech Perception and Production Tasks

Evidence that the neural processing of speech perception and production was altered in patients with RpSTS gliomas, compared to a large sample of neurotypical control participants and a large sample of control patients with gliomas affecting other parts of the cerebrum, was identified in the left and right temporal lobes. The left superior temporal cortex showed enhanced activation during auditory semantic matching but not object naming; and the right superior temporal cortex showed abnormally low activation during auditory semantic matching but enhanced activation during object naming. These atypical effects cannot be explained by patients failing to perform the fMRI tasks (Price and Friston, 1999) because (i) the fMRI tasks were, by design, not particularly challenging and patients had accuracy in the range of the controls and (ii) the analysis was based on correct trials only. Nor can they be explained by tumour related disruptions to the hemodynamic response (Jiang et al., 2010; Wang et al., 2012) because (i) we observed abnormal responses in the tumour free left cerebral hemisphere; and (ii) the abnormalities in the right superior temporal cortex observed were task-specific in all three patients. Additionally, these effects were not seen in any of the further 16 patients with gliomas within other parts of the cerebrum indicating this is not a sequela of a non-specific tumour-induced disruption to language networks. Alternative interpretations are considered below.

### Abnormal fMRI Activation During Auditory Semantic Matching

Auditory input to each ear normally results in bilateral responses in the auditory cortices because ascending sensory afferent fibers from the brainstem cochlear nuclei project to both ipsilateral and contralateral auditory cortices (Parker Jones and Schnupp, 2021). In the context of right postero-superior temporal tumours or ischemic damage, it is not surprising that we observed reduced activation during auditory processing in the right auditory cortex. Although this did not result in abnormally slow responses or low accuracy during the auditory semantic matching task, speech perception impairments might have been observed with more stringent assessments, particularly when stimuli are only presented to the left ear given prior evidence that the crossed connections between ascending sensory afferent fibers and contralateral auditory cortices are more effective and functionally more important (Kimura, 1961; Nakasato et al., 1997; Jafari et al., 2016). For example, Nakasato et al. (1997) demonstrated that the presence of a tumour in the superior temporal lobe delays the auditory evoked potential to a stimulus when it is presented in the ear contralateral to the tumour but not when it is presented in the ear ipsilateral to the tumour.

Plausibly, normal behavioural responses during auditory semantic matching in the context of abnormally low responses in the right auditory cortex could be explained by the exaggerated response we observed in the undamaged left superior temporal lobe in the patients with right postero-superior temporal gliomas. For example, the enhanced response in the left hemisphere during auditory semantic matching might reflect enhanced, selective attention to right ear processing in the left temporal lobe because of impaired right temporal lobe function (Hugdahl and Andersson, 1986). Neurophysiologically, this can be supported through the presence of both ascending cochlear afferents projecting to bilateral auditory cortices and the presence of cortico-fugal projections to subcortical structures (Suga and Ma, 2003) through which top-down modulation can occur (Yan and Suga, 1998; Zhang and Suga, 2000). In our patients, these top-down effects are likely to be strategic because enhanced left temporal activation was not observed during object naming, even though bilateral auditory cortices are involved in processing the sound of the spoken response. Enhanced left temporal lobe activation was also not observed in any of the stroke patients even though three had no detectable activation in their right auditory cortex during auditory semantic matching. Plausibly this is because most of these patients were imaged at least 2 years following the time of their stroke. Future fMRI studies of stroke patients with right temporal lobe infarcts are required to test whether left temporal lobe activation is enhanced during auditory semantic matching in the immediate period following the onset of damage rather than later on following recovery.

An alternative explanation of enhanced left temporal lobe activation is that it represents contralateral disinhibition (Bloom and Hynd, 2005) from the damaged right auditory cortex but this would not explain why left temporal activation was not enhanced during object naming or in any of the stroke patients who had damage to the right auditory cortex. As we were only able to study three patients with right postero-superior temporal lobe gliomas, further studies are needed to determine whether enhanced left temporal activation during speech perception tasks is a result of (a) different neuroplasticity mechanisms following gliomas and infarcts; or (b) other individual differences (e.g., strategic, functional anatomy) that are independent of pathology.

### Abnormal fMRI Activation During Object Naming

Given the reduction in the right pSTS response during auditory semantic matching, it is surprising that no such reduction was observed during the speech production task (object naming). To the contrary, RpSTS activation during object naming was either higher than normal (patients A,B) or in the high normal range (patient C). This enhanced RpSTS activation during object naming, but not during the auditory semantic matching task was also observed in stroke patients with right hemisphere infarcts affecting, but not destroying, the right postero-superior temporal lobe. In other words, it was observed across two pathologies that are associated with different onsets (slow vs. acute), neuroplasticity mechanisms (continuous in the context of actively progressing damage with growing tumours vs. time limited in the case of a suddenly occurring infarct).

The fact that right temporal lobe object naming activation is not reduced within tumour infiltrated auditory cortex is informative in three ways. The first is that, despite RpSTS being infiltrated by the tumours, its task-specific activation during object naming indicates that it could still respond during accurate (in-scanner) speech production. Second, this task-specific effect (under-activation during auditory semantic matching, enhanced activation during object naming) is unlikely to represent the sequela of a perturbed hemodynamic response function which has been described when functional imaging has been performed in patients with brain tumours (Ojemann et al., 1998; Jiang et al., 2010; Wang et al., 2012). To the contrary, the task-specific responses provide evidence that the RpSTS region is functionally active despite being infiltrated by tumour. This is in keeping with previous studies demonstrating preserved function within tumour infiltrated cortices using intra-operative mapping in both low (Duffau et al., 2003; Benzagmout et al., 2007) and high grade gliomas (Ojemann et al., 1996), magnetoencephalography (MEG) (Schiffbauer et al., 2001) and fMRI (Krainik et al., 2003). It is also in keeping with our observations that the same pattern of response (under-activation during auditory semantic matching and enhanced activation during object naming) was observed in patients with RpSTS gliomas and stroke patients with right postero-superior temporal lobe infarcts. Third, if activity within tumour infiltrated auditory cortex does support object naming, complete loss of RpSTS (e.g., after resection) may result in more object naming errors, especially given that deficits were already detectable on behavioural language testing in all three patients.

In terms of how RpSTS contributes to object naming, we have previously proposed that it is normally responsive to auditory bottom up inputs and top down attention to the expected auditory output during speech production (Yamamoto et al., 2019b). Both these observations implicate RpSTS in the auditory monitoring of one’s own spoken response, which is necessary to ensure that sounds produced by the subjects’ articulators correspond to the sounds they were intended to produce (Yamamoto et al., 2019b). This is consistent with the current observation that RpSTS is a speech perception area that responds during auditory semantic matching and object naming but not visual semantic matching (see cyan areas in Figure 2). Prior studies have also shown how RpSTS activation increases when the auditory feedback is perturbed during speech production creating a mismatch between heard speech and intended speech (Houde and Jordan, 1998; Tourville et al., 2008; Zheng et al., 2010; Niziolek and Guenther, 2013; Behroozmand et al., 2015).

According to this theoretical account of RpSTS function, the continued RpSTS activation in our patients with RpSTS damage, during object naming but not auditory semantic matching, might reflect the increased demands on a disrupted speech monitoring system that attempts to minimises error-prone speech by increasing attention to top-down predictions relative to the perturbed auditory inputs from their own speech (brought about by the tumour). Such a top-down processing mechanism is more effective during speech production than speech perception because the patients can predict the sounds they are about to produce but cannot so easily predict the auditory signals produced during speech perception tasks (Blank et al., 2018).

### Tumour-Related Plasticity in the Patients With Low and High Grade Gliomas

Our findings of altered fMRI activation that was common across all three patients may be surprising given the generally held view that more slowly growing low grade gliomas have the potential for neural plasticity (Duffau, 2006, 2005; Desmurget et al., 2007) while more aggressive high grade gliomas have limited potential for remodeling. On the other hand, there have been previous reports of both low and high grade gliomas inducing the same pattern of reorganisation in language networks. This has been demonstrated with intra-operative mapping (Lubrano et al., 2010), positron emission tomography (Thiel et al., 2001), repetitive transcranial magnetic stimulation (Thiel et al., 2005), language fMRI (Bizzi et al., 2012) and resting state fMRI (Briganti et al., 2012). Tumour grading alone is therefore not sufficient to predict the potential for tumour-related plasticity, which likely relates to a multitude of factors which include the tumour’s rate of growth, its size, location and which eloquent brain regions are infiltrated.

## Limitations and Future Directions

The main limitation of this study was the small cohort size of patients with right postero-superior temporal gliomas. We attempted to mitigate the reporting of spurious (subject specific) findings, by focusing on fMRI abnormalities that were consistent across all three participants despite differences in their age, tumour histology and size. We also demonstrate that the task specific responses in RpSTS were observed in stroke patients with right hemisphere infarcts that involved the right postero-superior temporal lobe. Nevertheless, future studies are required to determine how well our findings replicate over larger cohorts of patients with right postero-superior temporal gliomas, and any factors that influence functional reorganisation of language processing in these patients. Repeated longitudinal language testing would also help determine whether progressive language impairments in speech production tasks are accompanied by a reduction in compensatory RpSTS activation and whether this is accompanied by contra-lateral hemispheric functional reorganisation, as observed during speech perception. Additional studies will also be required post-operatively to determine the effect of the tumour resection on both language assessment and the pattern of fMRI activation. Whilst the effect on language function is not as severe as with left temporal lobe resection, decline in verbal learning and memory and letter fluency have still been observed in 31% and 26% of patients undergoing resection of right temporal gliomas (Noll et al., 2015b). The aim will be to determine if any post-operative decline in language function is influenced by the presence or absence of altered activation in right pSTS.

A second potential limitation is that abnormal fMRI responses may reflect dysfunctional neurovascular coupling. Previous studies performed in patients with gliomas have demonstrated reduced fMRI BOLD activation across the tumour affected hemisphere (Jiang et al., 2010; Wang et al., 2012). This has been proposed to be due to tumour-related perturbation of the normal brain environment leading to neurovascular uncoupling (Zacà et al., 2014) that impacts the assessment of language hemispheric dominance (Ulmer et al., 2004). An altered BOLD response resulting from close proximity of a glioma is however not always the case (Yamamoto et al., 2019a) and even high grade gliomas have been shown to retain the normal task-evoked response resulting in localised cerebral oxygenation alteration and preserved BOLD activation close to the tumour (Fujiwara et al., 2004). Additional MRI perfusion studies could help to assess whether perfusion is abnormal within and/or close to tumour-infiltrated parenchyma. Examination of resting state fMRI in the RpSTS region would also be informative. The atypical language activations observed in the current study are however unlikely to be the consequence of neurovascular uncoupling because the direction of the BOLD response in the tumour affected hemisphere varied between tasks (Figure 4). Future experiments are, nonetheless, required to investigate whether right postero-superior temporal intra-tumoural fMRI activation, during object naming, represents preserved intra-tumoural function. This could be achieved using a non-invasive technique such as navigated-TMS or could also be performed intra-operatively with electrical stimulation techniques for cortical mapping (Picht et al., 2013).

## Summary and Conclusion

The current study found evidence for mild language impairments and atypical language activation in patients with gliomas (low and high grade) infiltrating the right postero-superior temporal lobe. Neural differences that were consistent across patients, compared to neurotypical controls participants and glioma control patients, were observed both within the tumour infiltrated right temporal lobe and within the contra-lateral hemisphere, representing two of the previously proposed patterns of plasticity (Desmurget et al., 2007).

One of the most striking results was that atypical neural activation in all three patients was task specific with abnormally low RpSTS activation during auditory semantic matching that was not seen during object naming. Our explanation for these effects is as follows. During auditory semantic matching, the processing of auditory inputs to right auditory cortex is perturbed. The abnormally high activation we observed in the left auditory cortex for the patients with right temporal lobe gliomas but none of the eight patients with right temporal lobe stroke damage suggest individual differences in the strategic re-directing of attention to the left auditory cortex but further studies are required to confirm this. In contrast during object naming, activation with right postero-superior temporal damage increases rather than decreases in the RpSTS, a region which is implicated in speech production in the healthy brain (Tian and Poeppel, 2013, 2010; Tian et al., 2016; Yamamoto et al., 2019b). We propose that these results are being driven top-down, rather than bottom up, and reflect the special role that RpSTS plays in monitoring self-generated speech.

## Clinical Implications

Our findings have several clinical implications. First, to reduce the risk of post-operative language deficits it is important for neurosurgeons to understand which brain regions might be compensating for the function of the region affected by the tumour so that these compensatory regions can be preserved during resection (Duffau, 2006, 2005). In addition to performing routine clinical fMRI tasks which can help to identify interhemispheric lateralisation of language activation (Black et al., 2017; Bargalló et al., 2020) additional preoperative fMRI tasks could be tailored depending upon which particular brain regions are affected by the tumour, and what is known about the function of these regions in the neurologically intact brain. For tumours affecting the right postero-superior temporal lobe, given the pre-existing knowledge of the function of RpSTS and the findings from this study, we would recommend using a two-object naming task performed with overt speech production (as has been used in this study) which has high consistency of reproducible activation across participants (Ekert et al., 2021).

In addition to fMRI, both intra-tumoural and peri-tumoural functional centres could be further assessed pre-operatively with other non-invasive techniques such as MEG (Zimmermann et al., 2019) and navigated-TMS (Ille et al., 2015). This combined increased pre-operative knowledge of sites of language function could lead to modification of the surgical technique such as the decision to perform the surgery awake with intra-operative mapping of the language system, particularly for right hemispheric tumours which are more likely to be operated on under general anesthesia than those in the left hemisphere (Hervey-Jumper et al., 2015; Vilasboas et al., 2017). If atypical activation occurs in the contra-lateral hemisphere as was shown with speech perception, it may also be important to confirm that these regions have been recruited prior to surgery in order to minimise any post-operative deficit. It has been suggested that surgery performed for low grade gliomas, particularly in patients with no or only minimal deficits, should be performed awake irrespective of the hemisphere (Duffau, 2018). If pre-operative investigations, fMRI with corroborative evidence from MEG/TMS, do identify right intra- or peri-tumoural language centres then we would be in agreement with this recommendation, which should also be extended to include patients with higher grade tumours such as those reported in the current study.

Second, insight into the mechanisms which support speech perception and production could allow for better predictions of long term outcomes for future patients. For example, if we know which regions can compensate for loss of another region infiltrated with tumour, then recovery or maintenance of function will depend on whether the tumour has spared compensatory pathways.

Finally, our study motivates further pre-operative language fMRI studies of patients with right hemispheric tumours which has historically not been the case (Manan et al., 2020). These may further reveal how right sided gliomas alter language performance and reorganisation within the language network.

## Data Availability Statement

The raw data supporting the conclusions of this article are available upon request from the senior author CJP, c.j.price@ucl.ac.uk.

## Ethics Statement

The studies involving human participants were approved by the NHS Research Ethics Committee–London Bridge and the London Queen Square Research Ethics Committee. The patients/participants provided their written informed consent to participate in this study.

## Author Contributions

AKY and CJP conceived, designed the study, analysed and interpreted the tumour patient and control data, drafted the manuscript. AKY, RP, and LM acquired and processed the tumour patient data. AGV and MC were involved with the acquisition and analysis of the stroke patient data with CJP. JE was involved with the analysis of the patient fMRI data. AS, OPJ, TMHH, SP, and MO acquired and processed the control data. AS, OPJ, TMHH, SP, CJP, DWG, and MO designed and optimised the fMRI paradigms. TAY provided overview to the study. All authors contributed to the article and approved the submitted version.

## Conflict of Interest

The authors declare that the research was conducted in the absence of any commercial or financial relationships that could be construed as a potential conflict of interest.

## Publisher’s Note

All claims expressed in this article are solely those of the authors and do not necessarily represent those of their affiliated organizations, or those of the publisher, the editors and the reviewers. Any product that may be evaluated in this article, or claim that may be made by its manufacturer, is not guaranteed or endorsed by the publisher.

## Abbreviations

BOLD: blood oxygenation level dependent
fMRI: functional magnetic resonance imaging
LGG: low grade glioma WHO (World Health Organization) grade 1-2
GOI: glioma patients of interest
GC: glioma control patients
HGG: high grade glioma WHO (World Health Organization) grade 3-4
MEG: Magnetoencephalography
PET: Positron emission tomography
pSTS: posterior superior temporal sulcus
PT: planum temporale
RpSTS: right posterior superior temporal sulcus
SC: stroke control patients
SOI: stroke patients of interest
TMS: transcranial magnetic stimulation.
