# Supplementary material for: The Effect of Right Temporal Lobe Gliomas on Left and Right Hemisphere Neural Processing During Speech Perception and Production Tasks
Source: Front Hum Neurosci. 2022 May 16;16:803163. doi: 10.3389/fnhum.2022.803163 (PMC9148966; doi:10.3389/fnhum.2022.803163)
Supplement: Supplementary file 1 [file Data_Sheet_1.docx]

# Appendices

**Appendix 1**

Information about the glioma control patient (GC) group

| **Patient** | **Age** | **Gender** | **Hand**  **edness** | **Hemi**  **sphere** | **Lobe** | **WHO** | **Tumour**  **type** | **IDH** | **1p 19 q** |
| --- | --- | --- | --- | --- | --- | --- | --- | --- | --- |
| GC_1 | 34.9 | F | L | R | F | 2 | OA | IDH1 | N |
| GC_2 | 21.6 | M | R | R | P | 1 | PGT | N | N |
| GC_3 | 48.1 | M | R | L | T | 3 | AA | N | N |
| GC_4 | 52.4 | M | R | R | P | 2 | OA | IDH1 | 19q |
| GC_5 | 42.2 | F | R | L | I>F>T | 2 | OA | IDH1 | N |
| GC_6 | 50.2 | F | L | L | I>F>T | U | U | U | U |
| GC_7 | 36.8 | M | R | L | I>F>T | 2 | OD | IDH1 | 1p 19 q |
| GC_8 | 44.1 | F | R | R | F | 3 | AO | IDH1 | 1p 19q |
| GC_9 | 42.9 | M | R | L | T | 2 | OA | IDH1 | N |
| GC_10 | 58.5 | M | R | L | F | 2 | OD | IDH2 | 1p 19q |
| GC_11 | 44.5 | M | L | L | F | 2 | OD | IDH1 | 1p 19q |
| GC_12 | 41.2 | M | R | R | F | 3 | AOA | IDH1 | 19q |
| GC_13 | 26 | F | R | L | P | 2 | DA | IDH1 | 19q |
| GC_14 | 44.8 | F | R | L | P | 2 | OD | IDH1 | 1p 19q |
| GC_15 | 22.7 | F | R | R | T | 1 | GG | N | n/a |
| GC_16 | 33.2 | F | R | R | T | 2 | OD | IDH2 | Ip 19 q |

Age (in years) at scan. Gender F= female, M= male. Handedness R= right, L= left. Hemisphere R= right, L= left. Lobe: Frontal (F), Parietal (P), Temporal (T), Insula (I). WHO grade (1, 2, 3), U= unknown (no surgery). Tumour OA= oligoastrocytoma, PGT= papillary glioneuronal tumour, AA= anaplastic astrocytoma, OD= oligodendroglioma, AO= anaplastic oligodendroglioma, AOA= anaplastic oligoastrocytoma, DA= diffuse astrocytoma, GG= ganglioglioma. IDH= presence of an IDH mutation, N= not present. 1p 19q= presence of deletion, N= not present, n/a= not available. Tumour details are based upon the 2016 classification (Louis et al, 2016).

**Appendix 2**

Information about the stroke patients of interest (1-8) and the stroke control patients (9-16)

| **Group ID** | **PLORAS ID** | **Years post stroke** | **Age at scan** | **Gender** | **Handed**  **ness** | | **Hemisphere** | **Lesion size (cm^3^)** | **Lobes/structures affected** |
| --- | --- | --- | --- | --- | --- | --- | --- | --- | --- |
| SOI_1 | PS0576 | 14.2 | 63 | M | R | | R | 218 | Fronto-parieto-temporal, occipital (minor) |
| SOI_2 | PS1003 | 2.2 | 50 | F | R | R | | 151 | Fronto-temporo-parieto-insula, basal ganglia |
| SOI_3 | PS0402 | 4.9 | 60 | M | R | | R | 90 | Temporo-insula-frontal, basal ganglia |
| SOI_4 | PS0672 | 5.1 | 67 | F | L | | R | 219 | Fronto-temporo-parieto-insula, basal ganglia |
| SOI_5 | PS0607 | 5.0 | 68 | M | R | | R | 205 | Fronto-temporo-parietal, basal ganglia |
| SOI_6 | PS1066 | 1.9 | 59 | M | R | | R | 236 | Fronto-insula-temporo-parietal, basal ganglia |
| SOI_7 | PS0678 | 10.2 | 73 | M | R | | R | 238 | Fronto-temporo-parieto- insula, basal ganglia |
| SOI_8 | PS0596 | 3.1 | 45 | M | R | | R | 276 | Fronto-temporo-parieto-insula,  occipital (minor), basal ganglia |
|  |  |  |  |  |  | |  |  |  |
| **GroupID** | **PLORAS ID** | **Years post stroke** | **Age at scan** | **Gender** | **Handed**  **ness** | | **Hemisphere** | **Lesion**  **size (cm^3^)** | **Lobes/structures affected** |
| SC_1 | PS0398 | 15.4 | 72 | F | R | | R | 55 | Fronto-parietal white  matter, insula, basal ganglia |
| SC_2 | PS0706 | 6.7 | 54 | M | R | | R | 29 | Fronto-insula, basal ganglia |
| SC_3 | PS0708 | 2.1 | 55 | M | R | | R | 139 | Fronto-parieto-insula, basal ganglia |
| SC_4 | PS0790 | 4.5 | 49 | M | R | | R | 85 | Occipital, ventro-medial temporal, parietal |
| SC_5 | PS0904 | 2.7 | 56 | M | R | | R | 53 | Ventral occipito-temporal |
| SC_6 | PS1446 | 15 | 49 | F | R | | R | 93 | Fronto-insula, basal ganglia (minor) |
| SC_7 | PS2280 | 3.3 | 57 | M | R | | R | 11 | Basal ganglia |
| SC_8 | PS1190 | 0.8 | 26 | M | R | | R | 27 | Fronto-insula, parieto-temporal (minor), basal ganglia |

Gender F= female, M= male. Handedness R= right, L= left. Hemisphere R= right, L= left

**Appendix 3 (Image 1.tiff)**

Object naming activation in the 8 stroke patients with RpSTS damage. Structural images for each of the stroke patients of interest (SOI) with (i) damage around RpSTS (at +51, +25, +5) but (ii) signal at p<0.001 in the F-map for the first level fMRI analysis. For each patient, the left image is a sagittal section of the structural (x=+51) with crosshairs at y=-25 and z=+5; and the right image is the same with object naming activation (p<0.001) superimposed. The group results (Table 6) remained significant even when PS1066, PS0596 (with apparent movement artefacts) were removed).
