# Supplementary figures and images for: The Effect of Right Temporal Lobe Gliomas on Left and Right Hemisphere Neural Processing During Speech Perception and Production Tasks
Source: Front Hum Neurosci. 2022 May 16;16:803163. doi: 10.3389/fnhum.2022.803163 (PMC9148966; doi:10.3389/fnhum.2022.803163)

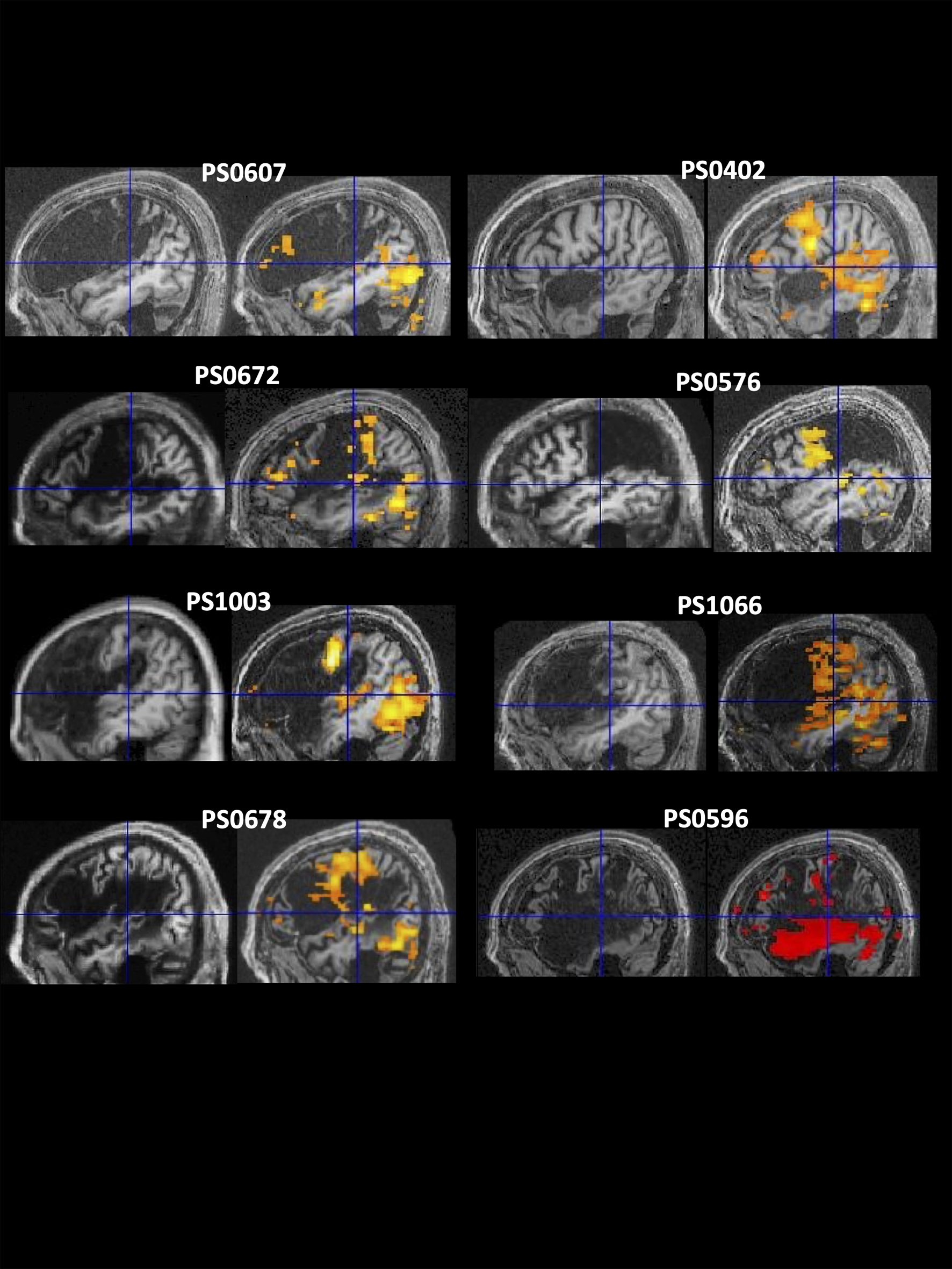

Supplement: Supplementary file 2 [file Image_1.TIFF]
